# Mechanical Support in Myocardial Infarction Complicated by Cardiogenic Shock: What Have We Learned from Trials?

**DOI:** 10.3390/jcm15124453

**Published:** 2026-06-09

**Authors:** Cristina Aurigemma, Norman Mangner, Vasileios Panoulas, Jacob Eifer Møller

**Affiliations:** 1 Department of Cardiovascular Sciences, Fondazione Policlinico Universitario A. Gemelli IRCCS, 00168 Rome, Italy; 2 Department of Internal Medicine and Cardiology, Herzzentrum Dresden, Faculty of Medicine, University Hospital Carl Gustav Carus, TUD Dresden University of Technology, 01307 Dresden, Germany; norman.mangner@tu-dresden.de; 3 Department of Cardiology, Harefield Hospital, Royal Brompton and Harefield Hospitals, Guy’s and St Thomas’ NHS Foundation Trust, London UB9 6JH, UK; vasileios.panoulas2@nhs.net; 4 Department of Cardiology, Copenhagen University Hospital, 2100 Copenhagen, Denmark; jacob.moeller1@rsyd.dk

**Keywords:** cardiogenic shock, myocardial infarction, Impella, intra-aortic balloon pump, VA-ECMO, microaxial flow pump

## Abstract

Cardiogenic shock (CS) is the most lethal complication of acute myocardial infarction (AMI), with a 30-day mortality of approximately 40–50% despite early revascularization. Temporary mechanical circulatory support (tMCS) devices, including the intra-aortic balloon pump (IABP), microaxial flow pumps (MAFP) and veno-arterial extracorporeal membrane oxygenation (VA-ECMO), are used as adjunctive therapy in refractory shock, but evidence of a survival benefit is limited and often conflicting. The IABP-SHOCK II trial found no 30-day mortality reduction with IABP, supporting a Class III (no benefit) recommendation, whereas the DanGer Shock trial reported a 12.7% absolute mortality reduction at 180 days with the MAFP Impella CP in highly selected patients. In contrast, the ECLS-SHOCK and ECMO-CS trials showed no improvement in survival with early VA-ECMO and noted high complication rates. Real-world data reveal significant disparities between trial populations and clinical practice, highlighting limitations of current evidence, since many AMI-CS patients are older, in more advanced shock or have multiple comorbidities and would not meet typical randomized controlled trial (RCT) inclusion criteria. In clinical practice, in-hospital mortality with IABP or VA-ECMO often exceeds 50–60%. Given the heterogeneity of AMI-CS, rapid identification of appropriate tMCS candidates and personalized therapy are essential. Management guided by individual patient profile, hemodynamic stage and neurological status, supported by multidisciplinary shock teams, may improve timely triage, device selection and outcomes. This review emphasizes the need for individualized, protocol-driven care within structured shock systems to optimize tMCS use in AMI-CS.

## 1. Introduction

Cardiogenic shock complicates approximately 5–10% of acute myocardial infarctions and remains highly lethal. Contemporary data indicate that acute myocardial infarction-related cardiogenic shock (AMI-CS) carries a 30-day mortality of around 40–50%, rising to more than 50% at one year despite aggressive management [1,2]. AMI-CS is not a uniform clinical entity. Approximately 40% of cases are characterized by predominant left ventricular (LV)-dominant pump failure (“classic” cardiogenic shock); around 50% occur in the setting of post-cardiac arrest with variable degrees of myocardial and neurological injury; and less than 10% are attributable to predominant right ventricular (RV) failure or mechanical complications such as ventricular septal defect or papillary muscle rupture [3,4,5]. These subgroups differ in pathophysiology, hemodynamic profiles and prognostic trajectories. Therefore, careful hemodynamic assessment and prompt imaging are critical to determine etiology and tailor therapy to each patient’s profile.

**Figure 1:**
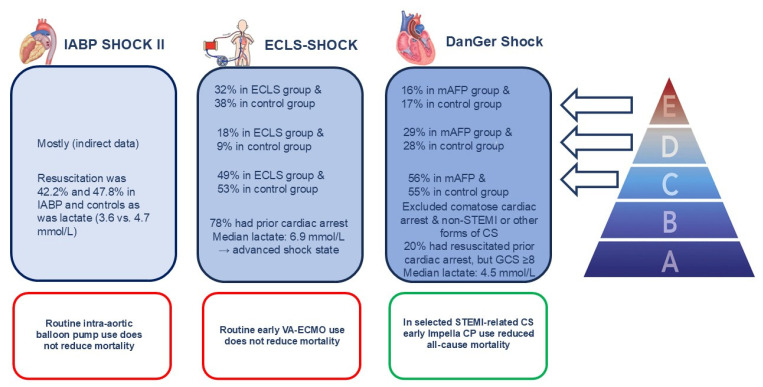
Summary of major randomized trials in AMI-related cardiogenic shock (IABP-SHOCK II, DanGer Shock, ECLS-SHOCK). The figure illustrates differences in patient features, study design and device strategies that limit the generalizability of results to broader shock populations. CS, cardiogenic shock; MAFP, microaxial flow pump; STEMI, ST-elevation myocardial infarction.

Early revascularization of the infarct-related artery remains the cornerstone of therapy and improves survival in AMI-CS [6]. Accordingly, current guidelines give a Class I B recommendation for immediate culprit-lesion revascularization in AMI-CS [7]. For non-culprit lesions, European and American guidance advise that routine immediate multivessel percutaneous coronary intervention (PCI) is not recommended in cardiogenic shock, given the potential for procedural complications, longer ischemic times and contrast overload [7,8]. However, even with prompt revascularization, many patients continue to experience profound hemodynamic instability and require additional supportive therapies. Pharmacologic support with vasopressors and inotropes is often necessary to maintain systemic perfusion. Norepinephrine is generally preferred as the first-line vasopressor because of lower arrhythmia risk compared with dopamine, and inotropic agents such as dobutamine, milrinone or adrenaline are commonly used to augment cardiac output [9]. In addition to pharmacotherapy, temporary mechanical circulatory support (tMCS) devices are increasingly used in refractory cardiogenic shock to improve hemodynamics. Options include the intra-aortic balloon pump (IABP), percutaneous ventricular assist devices such as the microaxial flow pump (MAFP, Impella^®^) and veno-arterial extracorporeal membrane oxygenation (VA-ECMO). In theory, these devices can unload failing ventricles or provide supplemental circulatory flow, serving as bridges to myocardial recovery or to more definitive interventions. However, evidence that tMCS improves survival outcomes remains mixed and often limited (Figure 1). The present review provides a critical analysis of the available evidence and current perspectives on the use of tMCS in AMI-related cardiogenic shock.

## 2. Intra-Aortic Balloon Pump in Cardiogenic Shock

### 2.1. Mechanism of Action

The IABP provides hemodynamic support through diastolic counterpulsation. A balloon catheter positioned in the descending thoracic aorta inflates during diastole, augmenting aortic diastolic pressure and improving coronary perfusion, and deflates just before systole, reducing aortic pressure and thereby decreasing LV afterload. This coordinated inflation–deflation cycle aims to reduce myocardial oxygen demand while increasing supply. However, the hemodynamic effect is modest and depends on residual LV function, typically increasing cardiac output by only ~0.5–1.0 L/min [10] (Table 1). In a randomized trial, IABP did not improve cardiac output or lower filling pressures compared with dobutamine in AMI-CS [10].

### 2.2. Clinical Evidence

Despite long-standing use in AMI-CS, randomized trials have not demonstrated a survival benefit. The IABP-SHOCK II trial (*n* = 600) randomized patients with AMI-CS undergoing early PCI to IABP therapy or standard medical management [11]. It found no reduction in 30-day mortality (39.7% vs. 41.3%, *p* = 0.69) and no benefit in secondary outcomes such as hemodynamic stabilization, lactate clearance or intensive care unit (ICU) length of stay [11] (Table 2). Crossover occurred in approximately 10% of patients, reflecting rescue IABP use in deteriorating controls; a total of 7.4% in the IABP arm and 5.1% in the standard-care arm required escalation to more advanced support. Excluding crossovers yielded the same neutral result. Long-term follow-up at six years confirmed no mortality benefit [12]. Safety outcomes were broadly comparable: major peripheral ischemia requiring intervention occurred in 4.3% versus 3.4% (*p* = 0.53) and life-threatening or severe bleeding in 3.3% versus 4.4% (*p* = 0.51). Overall, routine IABP did not improve clinical outcomes (Table 1).

### 2.3. Guideline Recommendations and Clinical Practice

Consistent with these data, international guidelines have downgraded IABP for AMI-CS. The 2023 ESC guidelines give a Class III (no benefit), Level A recommendation against routine IABP use in AMI-CS, while noting a potential role as a temporary bridge in mechanical complications (e.g., ventricular septal defect or papillary muscle rupture) [7]. The 2025 AHA/ACC statement similarly assigns Class III (no benefit) to routine IABP support in AMI-CS without mechanical complication, reserving Class IIb scenarios for refractory shock when more advanced devices are unavailable or when mechanical complications require urgent stabilization [8]. Thus, while IABP remains widely available, routine use in AMI-CS is no longer supported by evidence or guidelines (Table 1).

## 3. Microaxial Flow Pump in Cardiogenic Shock

### 3.1. Mechanism of Action

The Impella™ system is a catheter-mounted MAFP positioned across the aortic valve. It unloads the LV by drawing blood from the LV and expelling it into the ascending aorta. Depending on the model (e.g., CP or 5.5), it provides forward flow of approximately 3.5–5.5 L/min, reducing LV end-diastolic pressure and volume. The unloading decreases myocardial work and oxygen demand while maintaining systemic perfusion, providing greater hemodynamic support than IABP without increasing afterload [15] (Table 1).

### 3.2. Clinical Evidence

Evidence for Impella in AMI-CS has evolved, though limitations remain. Early randomized data, particularly the IMPRESS trial, were inconclusive. This small multicenter randomized controlled trial (RCT) enrolled 48 patients with severe ST-elevation myocardial infarction (STEMI)-CS, of whom 44 (~92%) had out-of-hospital cardiac arrest with profound hemodynamic and metabolic compromise [16]. Median return of spontaneous circulation (ROSC) time was 22 min and baseline lactate exceeded 8 mmol/L, indicating prolonged low-flow states and high risk of hypoxic brain injury. By comparison, IABP-SHOCK II enrolled patients with lower severity (median lactate ~4 mmol/L) and only ~45% post-arrest [11]. IMPRESS showed no survival benefit at 30 days or 5 years: ~46% versus ~50% mortality at 30 days (HR 0.96; *p* = 0.92) and 50% versus 63% at 5 years (RR 0.87; *p* = 0.65) [16,17]. Given its small sample size and the predominance of post-arrest patients, the trial was underpowered and applies mainly to this phenotype. In such settings, neurological injury and systemic ischemia–reperfusion injury may dominate prognosis, potentially limiting the impact of LV unloading on survival. More definitive evidence emerged from the DanGer Shock trial, a larger multicenter RCT that randomized 360 patients with STEMI-CS to MAFP plus standard care versus standard care alone [13]. Patients were treated very early, and MAFP was frequently inserted prior to PCI in high-volume centers using strict protocols. At 180 days, all-cause mortality was reduced by 12.7% (45.8% vs. 58.5%; HR 0.74; *p* = 0.04), corresponding to a number needed to treat (NNT) of approximately 8 for the primary endpoint of 180-day all-cause mortality. The survival benefit was sustained for up to ten years [18]. The NNT was even lower (~5) in patients younger than 77 years [19], and benefit was observed in both women and men [20]. However, the mortality reduction came with higher complication rates, including major bleeding, limb ischemia, sepsis and the need for renal replacement therapy, with an approximate number needed to harm (NNH) of 6 for the composite of serious device-related adverse events (severe bleeding, limb ischemia, sepsis and renal replacement therapy) (Table 2). Importantly, DanGer Shock largely excluded comatose post-arrest patients, so results cannot be generalized to AMI-CS with severe hypoxic brain injury or to shock phenotypes not represented in the trial. The trial also highlights that favorable outcomes were achieved in expert centers, emphasizing early deployment, procedural expertise and structured complication management. Observational studies and meta-analyses (including one pooling more than 10,600 patients) have reported higher crude mortality and bleeding risk with MAFP compared with IABP (57% vs. 46% mortality; OR 1.44) [21]. However, these analyses are limited by confounding and selection bias: sicker patients are more likely to receive higher-support devices, and registries often lack key measures such as lactate, SCAI stage and timing of implantation. Some sub-analyses suggest better outcomes when Impella is used very early, before cardiac arrest or before escalation to high-dose vasopressors [22]. An individual patient-data meta-analysis of randomized trials on tMCS in AMI-CS reported no reduction in 6-month all-cause mortality with early routine tMCS irrespective of device type, alongside higher rates of major bleeding and vascular complications; any survival benefit appeared confined to STEMI patients without risk of hypoxic brain injury [23]. In a registry cohort retrospectively classified by DanGer Shock eligibility, 30-day mortality was lower in DanGer Shock-like compared with DanGer Shock-unlike patients (48.4% vs. 70.2%; *p* < 0.001), and 180-day mortality was also lower (62.5% vs. 72.0%; *p* = 0.014) [24]. Collectively, these findings support the concept that benefit is driven by timing and patient selection rather than device use per se.

### 3.3. Guideline Recommendations and Clinical Practice

Guideline positions reflect evolving evidence. The 2023 ESC STEMI guidelines (published before DanGer Shock) assign Impella a Class IIb recommendation (may be considered) in refractory cardiogenic shock, particularly when used early and in experienced high-volume centers, and acknowledge its role for LV unloading alongside VA-ECMO [7]. The 2025 AHA/ACC consensus, published after DanGer Shock, differentiates by device and assigns Impella a Class IIa rating (reasonable to reduce mortality) for STEMI complicated by severe or refractory cardiogenic shock [8]. This Class IIa (rather than Class I) designation reflects the balance between the survival benefit demonstrated in DanGer Shock and the substantial device-related complication burden. Thus, Impella appears most appropriate in carefully selected early-stage STEMI-CS patients managed in experienced centers with standardized protocols.

## 4. Veno-Arterial Extracorporeal Membrane Oxygenation in Cardiogenic Shock

### 4.1. Mechanism of Action

VA-ECMO provides temporary full cardiopulmonary support by draining venous blood, passing it through an oxygenator and returning it to the arterial system. This enables high-flow support (typically 3–5 L/min) and gas exchange, supporting patients with biventricular failure, severe hypoxemia or during cardiac arrest. A key limitation is that retrograde arterial flow can increase LV afterload, raising wall stress, oxygen demand and pulmonary congestion unless active LV unloading is used (e.g., Impella, surgical vent or atrial septostomy). Without unloading, LV distension may contribute to thrombus formation, pulmonary edema and further myocardial injury [25] (Table 1).

### 4.2. Clinical Evidence

Randomized evidence for VA-ECMO in AMI-CS is limited to two trials and remains inconclusive. The ECLS-SHOCK trial randomized 417 patients with AMI-CS undergoing planned early revascularization to early VA-ECMO versus standard care [14]. Thirty-day mortality was nearly identical (47.8% vs. 49.0%; RR 0.98; *p* = 0.81) (Table 2), and results remained neutral at one year [26]. Interpretation is complicated by several factors: the enrolled population was very sick (over 60% SCAI stage D/E), ~78% had cardiac arrest prior to randomization with median ROSC of 20 min, and inclusion criteria did not incorporate echocardiographic variables, leading to inclusion of patients with LVEF > 35–40% or systolic blood pressure (SBP) > 120 mmHg. Although LV unloading was prespecified, it was not consistently implemented. Despite increasing interest in adjunctive LV unloading during VA-ECMO, there is no randomized evidence demonstrating clinical benefit of routine unloading in AMI-CS. In a secondary analysis of DanGer Shock, the combination of Impella and ECMO (“ECMELLA”) was associated with substantially higher bleeding risk: major bleeding (BARC type 3–5) occurred in 64.3% with ECMELLA versus 24.5% with Impella alone [27]. Observational studies suggest unloading may improve hemodynamics or short-term endpoints in selected scenarios, but have not shown clear long-term mortality reductions and consistently report higher complication burdens (bleeding, limb ischemia, hemolysis) [28,29]. In ECLS-SHOCK, major bleeding and vascular complications were more frequent with ECMO, with major bleeding occurring in 23.4% versus 9.6% (RR 2.44).

The ECMO-CS trial (*n* = 117) evaluated rapidly deteriorating cardiogenic shock and compared immediate ECMO with a selective/delayed ECMO strategy [30]. The composite endpoint (death, resuscitated cardiac arrest or crossover to another device) occurred at similar rates (HR 0.72; *p* = 0.21), providing no support for routine early ECMO. Meta-analyses of observational studies suggest a potential benefit in cardiac arrest or profound biventricular shock, but these signals are highly susceptible to selection bias and are accompanied by high rates of bleeding, stroke and limb ischemia [31,32,33,34]. Individual patient-data meta-analyses of randomized trials likewise show no overall mortality benefit with early routine temporary MCS and increased bleeding/vascular complications, with any benefit confined to highly selected subgroups [23,34]. To date, no large head-to-head RCTs compare VA-ECMO directly with Impella or IABP (Table 1).

A mechanistically distinct indication for VA-ECMO is extracorporeal cardiopulmonary resuscitation (eCPR), in which VA-ECMO is deployed during ongoing refractory cardiac arrest rather than to support an already established shock state. The supporting evidence is specific to this setting and remains mixed. The single-center ARREST trial randomized patients with refractory out-of-hospital cardiac arrest and shockable rhythms to early ECMO-facilitated resuscitation versus standard advanced cardiac life support and was stopped early for a survival benefit in the ECMO arm [35]. In contrast, the multicenter INCEPTION trial found no significant difference in 30-day survival with a favorable neurological outcome between eCPR and conventional CPR, underscoring the dependence of any benefit on rapid, protocolized implementation and stringent case selection [36]. Because eCPR addresses arrest physiology and hinges critically on low-flow time and neurological prognosis, its indications, candidate selection and outcomes differ from those of VA-ECMO used for AMI-CS; it is therefore best regarded as a separate, time-critical application confined to experienced eCPR systems.

### 4.3. Guideline Recommendations and Clinical Practice

Current guidelines do not support routine VA-ECMO in AMI-CS. Both the 2023 ESC and 2025 AHA/ACC documents emphasize that ECMO should be reserved for selected patients with truly refractory shock or during ongoing cardiac arrest (eCPR), and only in experienced centers [7,8]. The ESC assigns VA-ECMO a Class IIb recommendation in severe shock or arrest scenarios if instituted early with a plan for unloading. In contrast, the AHA/ACC assigns routine ECMO a Class III (not recommended) in AMI-CS [8], reflecting neutral RCT results and high complication rates. Given evidence of worse outcomes in low-volume centers [37], ECMO should be confined to experienced high-volume programs and used as rescue therapy after shock-team evaluation until subgroups most likely to benefit are better defined.

## 5. Limitations of the Evidence

Despite the growing use of tMCS, the evidence base is limited and heterogeneous. A central challenge is the broad spectrum of “cardiogenic shock” in presentation and prognosis and the lack of a uniform definition. The SCAI classification provides a framework to stratify severity from Stage B to Stage E [38], with a 2022 revision incorporating modifiers such as lactate level and cardiac arrest [39]. However, shock severity is dynamic, criteria can be applied inconsistently and SCAI staging has not been uniformly embedded in major randomized trials. Moreover, treating any out-of-hospital cardiac arrest as a Stage E modifier may oversimplify neurological risk and post-arrest trajectories [40].

tMCS trials have enrolled markedly different populations. IABP-SHOCK II included mostly Stage C patients (median lactate ~4 mmol/L; ~45% post-arrest) [12]. IMPRESS randomized a profoundly compromised cohort (~94% out-of-hospital cardiac arrest, lactate > 8 mmol/L, ROSC 22 min), implying high risk of hypoxic brain injury [17]. ECLS-SHOCK similarly enrolled 78% post-arrest patients [14]. Such differences likely influenced neutral outcomes and limit the generalizability of any single trial beyond its enrolled phenotype. Conversely, DanGer Shock focused on a selected STEMI-CS population and excluded patients at high risk of irreversible neurological injury. Only ~20% had any cardiac arrest (during transport or after arrival), and comatose out-of-hospital arrest patients were excluded. Impella was implanted very early and according to standardized protocols at experienced centers; however, complication rates remained substantial, emphasizing the need for expertise and optimal timing of early intervention.

Real-world registries reinforce concerns about external validity (Table 3). Schrage et al. reported that only a minority of contemporary shock patients would qualify for major trials: 24.4% for DanGer Shock and 55.3% for IABP-SHOCK II [41]. Similarly, in the Dresden Impella Registry, 28.4% of STEMI-CS patients fulfilled DanGer Shock eligibility criteria [24]. These findings highlight that RCTs often exclude older patients, those with prolonged arrests and those with major comorbidities. Notably, in the Dresden registry, 30-day mortality remained high in both trial-eligible and trial-ineligible patients, suggesting that current criteria may not fully identify those most likely to benefit from tMCS. In routine practice, patients are often older, more metabolically compromised and present later in the shock course. Observational data show that real-world mortality with IABP often exceeds 50%, higher than the 39.7% in IABP-SHOCK II [12,13,14,42]. ECMO-related mortality in registries can exceed 60%, above RCT rates. For Impella, reported mortality in practice (often 45–60%) is closer to trial data, possibly reflecting selective use in specialized centers (Table 4). Recent analyses have attempted to refine selection by identifying “DanGer-like” profiles, i.e., patients with less advanced shock and intact neurological function. Some meta-analyses and registry evaluations suggest that when ECMO is applied in DanGer-like patients, outcomes are better than in non-DanGer-like patients treated with ECMO, underscoring the importance of neurological status and early selection [24,34,41,42,43,44,45]. Overall, these data emphasize that one-size-fits-all interpretations of RCTs may not apply across the spectrum of AMI-CS and support individualized decisions integrating trial and real-world evidence.

## 6. Discussion

Cardiogenic shock remains highly lethal after AMI, with 30-day mortality around 40–50% and ~50% at one year despite advances in reperfusion and critical care. A major challenge is the heterogeneity of presentation and trajectory. The SCAI framework stratifies severity from Stage A to Stage E and, together with modifiers such as lactate and cardiac arrest, can support prognostication and management decisions. This heterogeneity is reflected in key tMCS trials. IABP-SHOCK II predominantly enrolled moderately severe shock (median lactate ~4 mmol/L; <50% cardiac arrest) [12], whereas IMPRESS and ECLS-SHOCK included largely post-arrest populations (IMPRESS: 94% post-arrest with lactate > 8 mmol/L [17]; ECLS-SHOCK: 78% post-arrest [14]). These baseline differences likely contributed to neutral outcomes and limit generalizability beyond the enrolled phenotypes.

DanGer Shock stands out for its stringent selection and early intervention. By excluding patients at high risk of irreversible neurological injury, only ~20% had any cardiac arrest. Impella was initiated early, often before PCI, under standardized protocols at experienced centers. The trial demonstrated a 12.7% absolute reduction in 180-day mortality (45.8% vs. 58.5%; NNT~8) [13] with sustained benefit, but with higher rates of device-related complications (NNH~6). Observations that patients receiving Impella before reperfusion fared better support the hypothesis that LV unloading prior to PCI may improve myocardial recovery [15,22]. These data suggest that the “right patient” and “right timing” are central to achieving net benefit.

VA-ECMO offers full cardiopulmonary support but introduces unique physiological challenges. Retrograde arterial flow increases afterload and can worsen LV distension and ischemia unless unloading is implemented. Trials reported limited and inconsistent venting, potentially contributing to a lack of mortality benefit. Additionally, ECMO complication rates, particularly bleeding and vascular injury, are high and may offset hemodynamic gains in broad shock populations [25,32].

Guidelines reflect this mixed evidence (Table 1). The 2023 ESC guidelines provided a cautious Class IIb recommendation for Impella in refractory shock at experienced centers and advised against routine IABP or VA-ECMO (Class III) in AMI-CS [7]. These recommendations were formulated before the DanGer Shock and ECLS-SHOCK results, relying on earlier evidence [25,30]. In 2025, the ACC/AHA upgraded Impella to Class IIa for STEMI with severe or refractory shock while continuing to discourage routine VA-ECMO (Class III) [8]. Notably, no tMCS device has a Class I endorsement, underscoring continued uncertainty regarding optimal selection, timing and risk–benefit balance. Device-related bleeding and vascular risks, particularly outside expert centers or without established protocols, further justify caution.

Bridging the gap between trials and practice remains difficult. Many real-world patients (older, with delayed presentation and comorbidities) do not meet strict trial criteria such as those of DanGer Shock [24,41,42]. Registry studies show persistently high mortality (>50%) with MCS in AMI-CS regardless of trial eligibility, suggesting that current criteria may not fully capture determinants of benefit and futility. An individualized, protocol-driven approach is therefore essential. Multidisciplinary “shock teams” can expedite diagnosis, integrate hemodynamic and imaging data and coordinate early decisions about revascularization, pharmacotherapy and MCS [46]. Systematic reviews suggest that shock-team care is associated with higher 30-day and in-hospital survival and reduced ICU mortality compared with usual care [47]. Such teams bring together interventional cardiologists, heart failure specialists, intensivists, surgeons and others to standardize triage, select devices and manage complications, while avoiding futile interventions when recovery is unlikely.

**Figure 2:**
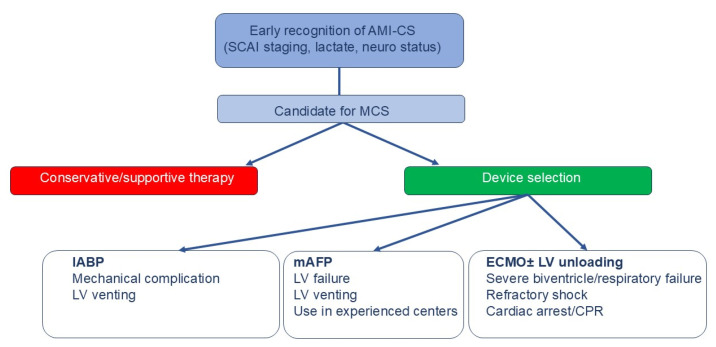
Proposed decision-making algorithm for mechanical circulatory support in AMI-related cardiogenic shock. Early recognition with SCAI staging, lactate levels and neurological status guides candidate selection. Device choice should be tailored to clinical profile and institutional expertise: IABP only for mild shock or LV venting when other options are unavailable; Impella for isolated LV failure in experienced centers; and VA-ECMO (with LV unloading when possible) for refractory shock, cardiac arrest or severe biventricular/respiratory failure. AMI-CS, acute myocardial infarction-related cardiogenic shock; IABP, intra-aortic balloon pump; LV, left ventricle; MAFP, microaxial flow pump; SCAI, Society for Cardiovascular Angiography and Interventions; VA-ECMO, veno-arterial extracorporeal membrane oxygenation.

In summary, early recognition of cardiogenic shock and timely hemodynamic support are crucial to improve prognosis by enabling intervention before irreversible metabolic or multiorgan injury. For patients with severe but potentially survivable shock (e.g., SCAI Stage C or D with ongoing hypoperfusion but intact neurological status), judicious early tMCS may stabilize perfusion and bridge to recovery or definitive therapy. Device choice should be tailored: IABP may be sufficient for LV venting or mechanical complications; Impella can support isolated LV failure; and VA-ECMO is appropriate for patients in extremis or with severe biventricular/respiratory failure, ideally combined with unloading when needed (Figure 2). Registry data also highlight that expertise matters: higher-volume CS/MCS centers have lower in-hospital mortality, with hazard ratios of approximately 0.80 for MCS-treated patients in centers performing ≥25 cases/year [37]. Improving survival in AMI-CS will likely require advances in devices and techniques, refined selection and timing, reduction in complications and shock-team pathways that deliver the right therapy to the right patient at the right time.

## 7. Conclusions

AMI complicated by cardiogenic shock remains associated with unacceptably high mortality despite modern early revascularization and advanced critical care. tMCS devices such as IABP, Impella and VA-ECMO can provide important hemodynamic support, but robust evidence for improved survival is limited and sometimes conflicting. Current data support the selective use of Impella in carefully chosen patients, whereas routine use of IABP or VA-ECMO in unselected AMI-CS is not supported. The DanGer Shock trial offers encouraging evidence that early LV unloading can improve outcomes in selected STEMI-CS patients, but the associated complication burden and narrow eligibility criteria limit broad applicability. Implementation of multidisciplinary cardiogenic shock teams has been linked to better outcomes, with systematic reviews reporting that shock-team care is associated with higher 30-day and in-hospital survival and reduced ICU mortality compared with usual care [47]. These findings support the use of coordinated team-based protocols in managing AMI-related shock. A multidisciplinary shock-team approach, guided by SCAI staging and early identification of patients most likely to benefit, appears central to improving care. Ongoing and future research should focus on refining the timing of MCS initiation, sharpening patient selection criteria and developing strategies to minimize complications, so that mechanical support can be better tailored to the needs of this diverse, high-risk population.

## Figures and Tables

**Table 1:** Percutaneous mechanical circulatory support devices in acute myocardial infarction-related cardiogenic shock.

	IABP	Impella	VA-ECMO
Mechanism of Action	Diastolic counterpulsation: Balloon inflates in diastole (↑ coronary perfusion), deflates before systole (↓ afterload). Modest ↑ in CO (~0.5–1.0 L/min). Minimal/no direct effect on RV.	Catheter-mounted microaxial pump placed across the aortic valve. Draws blood from LV to ascending aorta. Provides 3.5–5.5 L/min flow depending on model (CP, 5.5). Reduces LVEDP and myocardial O_2_ demand; maintains systemic perfusion. Indirect benefit on RV via LV unloading and reduced LVEDP.	Veno-arterial bypass: Venous drainage, oxygenation, arterial return. High-flow (3–5 L/min). ↑ LV afterload unless vented. Full cardiopulmonary support and full RV support (venous drainage and systemic return).
Introducer Size (French)	Typically 8 Fr.	Typically 14 Fr.	Typically 17–21 Fr venous; 15–19 Fr arterial.
Key Clinical Trial	IABP-SHOCK II (*n* = 600): AMI-CS patients post-PCI randomized to IABP vs. standard care.	IMPRESS (*n* = 48); DanGer Shock (*n* = 360).	ECLS-SHOCK (*n* = 417); ECMO-CS (*n* = 117).
Primary Outcome	IABP 39.7% vs. control 41.3% (*p* = 0.69). No mortality benefit.	IMPRESS: 30-day mortality 50% vs. 63% (*p* = 0.65); high-risk cohort (60% post-arrest, median lactate > 8 mmol/L). DanGer: Mortality 45.8% vs. 58.5% at 6 months; NNT~8; HR at 10 years 0.70 (*p* = 0.01).	ECLS-SHOCK: 47.8% vs. 49.0%; ECMO-CS: composite endpoint similar.
Other Findings	10% crossover from control to IABP. Escalation to advanced MCS: 7.4% (IABP) vs. 5.1% (control). No significant difference in vascular complications.	DanGer: 1.7% crossover, 21% protocolized escalation; ↑ complications (bleeding, limb ischemia, infection, RRT); NNH~6.	ECLS-SHOCK: ↑ bleeding (23.4% vs. 9.6%) and vascular complications. Venting inconsistently applied. Meta-analyses: possible benefit in arrest/biventricular failure but high complications.
Guideline Recommendations	ESC 2023 [7]: Class III (No Benefit) for routine use; consider in mechanical complications. AHA/ACC 2025 [8]: Class III for routine use; Class IIb in refractory shock or mechanical complications.	ESC 2023 [7]: Class IIb in refractory shock at expert centers; Class III for routine use. AHA/ACC 2025 [8]: Class IIa in STEMI with severe/refractory shock post-DanGer.	ESC 2023 [7]: Class III for routine use; IIb in refractory shock/arrest at expert centers. AHA/ACC 2025 [8]: Class III for routine use.

Abbreviations: AMI-CS, acute myocardial infarction-related cardiogenic shock; CO, cardiac output; CP, Impella CP; IABP, intra-aortic balloon pump; LV, left ventricle; LVEDP, left ventricular end-diastolic pressure; MCS, mechanical circulatory support; NNH, number needed to harm; NNT, number needed to treat; PCI, percutaneous coronary intervention; RRT, renal replacement therapy; RV, right ventricle; STEMI, ST-elevation myocardial infarction; VA-ECMO, veno-arterial extracorporeal membrane oxygenation. Symbols: ↑, increase; ↓, decrease.

**Table 2:** Comparison of major trials on mechanical circulatory support in acute myocardial infarction-related cardiogenic shock.

Trial (Year)	Population (*n*)	Device vs. Control	Primary Endpoint	Mortality Outcome	Major Complications
IABP-SHOCK II (2012) [11]	AMI-CS post-PCI (*n* = 600)	IABP vs. medical therapy	30-day mortality	39.7% vs. 41.3% (RR 0.96, *p* = 0.69)—no benefit	No difference in major bleeding or stroke; IABP safe but no hemodynamic advantage.
DanGer Shock (2024) [13]	STEMI-CS pre/post-PCI (*n* = 355)	Impella CP vs. medical	180-day mortality	45.8% vs. 58.5% (HR 0.74, *p* = 0.04)—↓ mortality	↑ Bleeding 22% vs. 12%; limb ischemia 5.6% vs. 1.1%; RRT 42% vs. 27%; sepsis ↑ (12% vs. 5%).
ECLS-SHOCK (2023) [14]	AMI-CS + PCI planned (*n* = 417)	VA-ECMO vs. medical	30-day mortality	47.8% vs. 49.0% (RR 0.98, *p* = 0.81)—no benefit	↑ Bleeding 23% vs. 10%; vascular injury 11% vs. 4%; longer ventilatory support with ECMO.

Abbreviations: AMI-CS, acute myocardial infarction-related cardiogenic shock; HR, hazard ratio; IABP, intra-aortic balloon pump; PCI, percutaneous coronary intervention; RR, risk ratio; RRT, renal replacement therapy; STEMI, ST-elevation myocardial infarction; VA-ECMO, veno-arterial extracorporeal membrane oxygenation. Symbols: ↑, increase; ↓, decrease.

**Table 3:** Main clinical features of trials and real-world registries of mechanical circulatory support in acute myocardial infarction-related cardiogenic shock.

Characteristic	DanGer Shock	Other RCTs (ECLS-SHOCK, ECMO-CS)	Real-World Registry
Patient selection	Highly selected	Moderately selected	Broad, less selected
Mean age	67 years	Similar	≈66 years
STEMI only	Yes	Mixed STEMI/NSTEMI	Mainly STEMI, variable
Pre-Impella CPR	20%	≈50% (ECMO-CS)	≈44%
LV ejection fraction	24%	30%	≈26.5%
Lactate levels	5.7 mmol/L	>5 mmol/L common	≈5.7 mmol/L
Device used	Impella CP	ECLS/ECMO	Mainly Impella CP, mixed with others
Timing of device	57% pre-PCI (early)	Variable	Often post-PCI
Standardization	Strict protocol	Variable across centers	Clinician judgement
PCI performed	97.6%	80–95%	88.9%
Additional MCS used	Yes, protocolized	Yes, depending on trial	Frequent, often combined

Abbreviations: CP, Impella CP; CPR, cardiopulmonary resuscitation; ECMO, extracorporeal membrane oxygenation; LV, left ventricle; MCS, mechanical circulatory support; NSTEMI, non-ST-elevation myocardial infarction; PCI, percutaneous coronary intervention; RCT, randomized controlled trial; STEMI, ST-elevation myocardial infarction.

**Table 4:** Outcomes of trials and real-world registries on mechanical circulatory support in acute myocardial infarction-related cardiogenic shock.

Parameter	Real-World	DanGer Shock	ECLS-SHOCK	IABP-SHOCK II
Total patients	528	355	417	600
Mortality with Impella	43%	45% (Impella group)	Not applicable	Not applicable
Mortality with VA-ECMO	63%	Not applicable	47.8% (VA-ECMO group)	Not applicable
Mortality with IABP	55%	Not applicable	Not applicable	39.7%
Revascularization rate	92%	>95%	~95%	100%
Use of MCS	33%	Impella vs. standard care	VA-ECMO vs. standard care	IABP vs. no IABP
Impella use	17%	Yes (randomized)	No	No
VA-ECMO use	9%	No	Yes (randomized)	No
IABP use	7%	No	No	Yes (randomized)

Abbreviations: IABP, intra-aortic balloon pump; MCS, mechanical circulatory support; VA-ECMO, veno-arterial extracorporeal membrane oxygenation.

## Data Availability

No new data were created or analyzed in this study.

## Abbreviations

The following abbreviations are used in this manuscript:

AMI	Acute Myocardial Infarction
AMI-CS	Acute Myocardial Infarction-related Cardiogenic Shock
CS	Cardiogenic Shock
tMCS	Temporary Mechanical Circulatory Support
MCS	Mechanical Circulatory Support
IABP	Intra-Aortic Balloon Pump
IABP-SHOCK II	Intra-Aortic Balloon Pump in Cardiogenic Shock II (trial)
MAFP	Microaxial Flow Pump
Impella	Microaxial Flow Pump (device brand/system)
VA-ECMO	Veno-Arterial Extracorporeal Membrane Oxygenation
ECMO-CS	Extracorporeal Membrane Oxygenation in Cardiogenic Shock (trial)
ECLS-SHOCK	Extracorporeal Life Support in Cardiogenic Shock (trial)
LV	Left Ventricle
RV	Right Ventricle
LVEDP	Left Ventricular End-Diastolic Pressure
PCI	Percutaneous Coronary Intervention
STEMI	ST-Elevation Myocardial Infarction
ROSC	Return of Spontaneous Circulation
RCT	Randomized Controlled Trial
NNT	Number Needed to Treat
NNH	Number Needed to Harm
HR	Hazard Ratio
RR	Risk Ratio
OR	Odds Ratio
ICU	Intensive Care Unit
BARC	Bleeding Academic Research Consortium
eCPR	Extracorporeal Cardiopulmonary Resuscitation
ECMELLA	Combined ECMO + Impella strategy
